# Effect on physiological parameters and anaesthetic dose requirement of isoflurane when tramadol given as a continuous rate infusion vs a single intravenous bolus injection during ovariohysterectomy in dogs

**DOI:** 10.1371/journal.pone.0281602

**Published:** 2023-02-08

**Authors:** Giovanna L. Costa, Simona Di Pietro, Claudia Interlandi, Fabio Leonardi, Daniele Macrì, Vincenzo Ferrantelli, Francesco Macrì

**Affiliations:** 1 Department of Veterinary Sciences, University of Messina, Messina, Italy; 2 Department of Veterinary Sciences, University of Parma, Parma, Italy; 3 Zooprophylactic Institute, Palermo, Italy; Stanford University School of Medicine, UNITED STATES

## Abstract

**Background:**

Tramadol produces a significant reduction in both sevoflurane and isoflurane minimum alveolar concentrations in dogs under experimental conditions. This study aims to compare the effects of tramadol administered as a constant rate infusion (CRI) with those of tramadol administered as a single intravenous bolus on physiological parameters and isoflurane requirements in dogs undergoing ovariohysterectomy.

**Methods:**

In this study, forty female dogs undergoing ovariohysterectomy were enrolled. The bitches were anesthetized with 5 mg/kg of tiletamine/zolazepam combined with 0.05 mg/kg of acepromazine intravenously. Anesthesia was maintained with isoflurane delivered in 100% oxygen. The group A (n = 20) received tramadol 4 mg/kg in a single intravenous bolus, whereas the group B (n = 20) received tramadol 1.5 mg/kg in an intravenous bolus followed by tramadol 2.6 mg/kg/h as a CRI. The following parameters were recorded: heart rate, respiratory rate, non-invasive blood pressure, body temperature, EtCO_2_, SpO_2_ and inspired and expired concentrations of isoflurane. Parameter measurements were performed from pre-preedication (baseline) to skin suturing.

**Results:**

The dogs were healthy subjects that demonstrated no abnormalities on laboratory investigations. Significant tachycardia was recorded after administration of tiletamine/zolazepam combined with acepromazine in both groups. Heart rate decreased after intubation but remained significantly higher compared to baseline values in both groups. Systolic blood pressure significantly decreased in both groups but the recorded values were within the physiological range. Mild reduction in body temperature was recorded in both groups. SpO_2_ and EtCO_2_ remained within the physiological range. Isoflurane requirement was significantly lower in the group B compared to the group A. Transient twitching was recorded in two dogs belonging to the group A after tramadol administration.

**Conclusions:**

Compared to tramadol given as a single intravenous bolus injection during ovariohysterectomy in dogs, tramadol administered as a CRI reduces isoflurane requirements in dogs anesthetized with tiletamine/zolazepam combined with acepromazine. Both tramadol given as a CRI and a single intravenous bolus injection, induce decrease in heart rate, respiratory rate and in body temperature but the values of these parameters remain within physiological range in dogs undergoing ovariohysterectomy.

## Introduction

Tramadol is a synthetic opioid with antinociceptive and analgesic effects due to opioid and non-opioid mechanisms of action. Tramadol consists of a racemic mixture of two enantiomers. The positive enantiomer has a higher affinity for the μ-opioid receptors and inhibits the reuptake of serotonin. The negative enantiomer inhibits the reuptake of noradrenaline whereas it is less effective on opioid receptors and serotonergic system [1]. Furthermore, one of its metabolites, O-desmethyltramadol, has weak μ agonist effects [2]. Nevertheless, tramadol may cause various side effects such as cardiorespiratory depression, reduction in gastrointestinal motility, dysphoria, and muscle twitching [3,4]. In dogs, tramadol was found to have a distribution half-life of 0.32 hour and an elimination half-life of 1.80 hour following intravenous (IV) administration of 4 mg/kg [5]. This rapid elimination rate underscores that dogs may require more frequent doses or a constant rate infusion (CRI) to maintain adequate therapeutic drug concentrations [5]. Previous studies demonstrated that both a single IV bolus and a CRI of tramadol produce a significant reduction in minimum alveolar concentrations of sevoflurane and isoflurane in dogs under experimental conditions [2,6,7]. The purpose of the present study was to compare the effects of a single IV bolus of tramadol to those of a CRI of tramadol on physiological parameters and isoflurane requirements in dogs undergoing ovariohysterectomy.

## Materials and methods

This study was approved by the Review Board for Animals Care of the University of Messina (protocol number 064–2021). Procedures were performed in accordance with Italian Law (D.M. 116192), Europe Law (O.J. of E.C. L 358/1 12/18/1986), and USA Laws (Animal Welfare Assurance No A5594-01, Department of Health and Human Services, USA). Prior to the dogs’ enrolment in the study, the owners provided a written informed consent.

In this study, forty female mixed breed dogs, aged 1.5 ± 0.5 years, weighing 16.5 ± 1.2 kg, were enrolled. We enrolled young normal healthy bitches to minimize individual variability as a source of error. The patients were referred for ovariohysterectomy. The dogs were subjected to physical examination and laboratory investigations (packed cell volume, total protein, albumin, alanine transaminase, aspartate transaminase, alkaline phosphatase, gamma-glutamyl transferase, total bilirubin, creatinine, urea nitrogen, glucose, and pH).

The patients were randomly divided into two groups (group A and group B), of twenty dogs each by drawing a ticket, after confirmation of their health status by both general clinical examination and laboratory investigations.

All animals were acclimated to the new environment for 45 minutes prior to the measurement of the following parameters (baseline values): heart rate (HR; beats per minute) was measured by auscultation using a stethoscope; respiratory rate (RR; breaths per minute) was measured by counting thoracic wall excursions; non-invasive blood pressure (mmHg) (systolic, SAP; mean, MAP; diastolic, DAP) by placing a small size cuff around the base of the tail and body temperature (°C) were measured using a monitor (Leonardo model, AMI Italia Srl, Milan, Italy).

A 20-G venous catheter was inserted in the right cephalic vein for medication and fluid administration. Tiletamine/zolazepam (Zoletil 10%, Virbac, Carros Cedex, France, 5 mg/kg) combined with acepromazine (Prequillan 1%, Fatro, Milan, Italy, 0.05 mg/kg) have been IV administered in all animals. Endotracheal intubation was performed with a cuffed tube. Anesthesia was maintained with isoflurane (Isoflo, Esteve, Milan, Italy) delivered in 100% oxygen via a non-rebreathing circuit (MATRX VMS, Alcyon, Cherasco, Italy).

Immediately after endotracheal intubation, a single IV bolus of tramadol (Altadol, Formevet, Milan, Italy, 4 mg/kg) has been administered in dogs belonging to the group A; an IV bolus of tramadol (1.5 mg/kg) followed by a CRI of tramadol (2.6 mg/kg/h) using a syringe pump (Compact Syringe Driver, Braun, Melsungen, Germany) has been administered in dogs belonging to the group B.

HR, RR, end-tidal carbon dioxide tension (EtCO_2_), arterial hemoglobin oxygen saturation (SpO_2_), body temperature, SAP, MAP, DAP, and the concentration of inspired and expired isoflurane were measured using a multiparametric monitor (Leonardo model). These parameters were recorded at the following time points: baseline values (before premedication B); at 5 minutes after administration of tiletamine/zolazepam combined with acepromazine (SD) (except EtCO_2_ and concentration of inspired and expired isoflurane); after endotracheal intubation (I); at the time of the skin incision (IC); during the traction of the first ovarian pedicle (TPI; right); during the traction of the second ovarian pedicle (TPII; left); at the time of the ligature of uterine body just cranial to the cervix (LC), at the beginning of the suture of the peritoneum (SP); and at the skin suturing (SC).

Intraoperative pain was assessed using a cumulative pain scale [8]. A numeric score between 0 and 4 was assigned based on the percentage variation from baseline values for RR, HR, and SAP at the aforementioned time points according to the following scheme: 0 = variation ≤ 0%; 1 = variation ≤ 10%; 2 = variation > 10% but ≤ 20%; 3 = variation > 20% but ≤ 30%; 4 = variation > 30%. The sum of the scores for the three parameters was the cumulative pain score (CPS). When the CPS was ≥ 10, the dog received 2.5 μg/kg of fentanyl IV (Fentadon, Dechra, Turin, Italy) as rescue analgesia.

Same individuals were assigned to collect data from the patients and a single anesthetist performed anesthesia for all the patients. The same surgeon performed all surgical procedures.

### Statistical analysis

Statistical analysis was performed using SPSS 15.0 (IBM Company, Milan, Italy). Shapiro-Wilk test was performed. Data with normal distribution were reported as mean +/- standard deviation (SD). CPS and data with no-normal distribution were reported as median and range. The differences along the time line were evaluated using Wilcoxon Test for data with no-normal distribution, and paired samples T test for data with normal distribution. The differences between groups were evaluated using Mann-Whitney U test for data with no-normal distribution, and independent samples T test for data with normal distribution. Statistical significance was set at p < 0.05.

## Results

The dogs were docile and healthy subjects that demonstrated no abnormalities on physical examination and had normal packed cell volume and biochemical parameters.

All the patient data collections, preforming anesthesia and surgical procedure was done by a fixed group of individual/s.

Surgical procedure lasted 10 ± 0.5 minutes in both groups.

Significant tachycardia was recorded in both groups at 5 minutes after administration of tiletamine/zolazepam combined with acepromazine. Heart rate decreased after intubation but remained significantly higher compared to baseline values in both groups. In the group B, heart rate significantly decreased were recorded during the traction of both ovarian pedicles, at the time of the ligature of the uterine body, at the beginning of the suture of the peritoneum, and at the skin suturing. HR was significantly higher in the group A compared to the group B (p = 0.020) [Table 1].

**Table 1:** Physiological parameters.

		Time								
Variables	Groups	B	SD	I	IC	TPI	TPII	LC	SP	SC
HR (beats minute)	A B	136 131/141 144 140/148	220 218/222* 223 219/229*	159 156/162* 165 161/169*	170† 163/177* 156 152/160*	171† 169/173* 130 125/135*	168† 166/170* 102 98/106*	157† 154/160* 94 91/97*	133† 129/137 104 100/108*	130† 125/135 102 98/106
RR (breaths minute)	A B	40 38/42*† 20 18/22*	28 25/31*† 9 7/11*	11 10/12* 11 9/13*	22 21/23* 5 3/7*	8 7/9* 3 2/4*	4 3/5* 5 2/8*	4 3/5* 5 3/8*	8/ 7/9* 9/ 5/13*	8 7/9* 10 8/12*
SAP (mmHg)	A B	140 138/142† 163 142/184	132 130/134*† 141 128/154*	128 126/130*† 168 155/184	130 128/132* 125 115/135*	135 134/136* 168 179/159	137 136/138* 125 117/137*	135 134/136* 137 125/149*	137 135/139* 140/ 132/148*	130 129/131*† 122 110/135*
MAP (mmHg)	A B	70 65/65† 90 80/100	73 69/76*† 70 62/80*	92 90/95*† 148 142/148*	98 95/102*† 105 97/112*	109 105/112* 133 125/140*	105 102/110* 100 90/109*	108 105/112*† 94 82/104*	107 100/111*† 68 60/64*	104 100/109*† 70 62/80*
DAP (mmHg)	A B	83 78/86 119 110/126	56 52/60*† 82 74/90*	80 72/80*† 135 130/146*	79 74/84† 89 82/96*	87 82/90*† 100 90/112*	85 82/88*† 74 62/76*	86 80/92*† 71 62/80*	85 80/90† 54 49/62*	84 80/87† 53 44/62*
EtCO2 (mmHg)	A B			38 36/40 38 36/40	46/ 39/51* 46 39/51*	44 41/47* 44 41/47*	44† 39/49* 44 39/49*	57 55/59* 57 55/59*	56 54/58* 56 54/58*	54 52/56* 54 52/56*
Sp02 (%)	A B		100±0 100±0	100±0 100±0	100±0 100±0	100±0 99±1	100±0 99±1	99±1 100±0	100±0 98±2	100±0 99±1
T (C°)	A B	39±0 39±0	39±0 39±0	39±0 39±0	39±0 39±0	39±0 39±0	38±0.2* 38±0.4*	38±0.5* 38±0.2*	38±0.2* 38±0.4*	38±0.5* 38±0.2*

HR = heart rate; RR = respiratory rate; SpO_2_ = arterial hemoglobin oxygen saturation; SAP = systolic non-invasive blood pressure; MAP = mean non-invasive blood pressure; DAP = diastolic non-invasive blood pressure; EtCO_2_ = end-tidal carbon dioxide tension; T = body temperature. B = baseline values (Before premedication); SD = 5 minutes after administration of tiletamine/zolazepam combined with acepromazine; I = after endotracheal intubation; IC = skin incision; TPI = traction of the first ovarian pedicle (right); TPII = traction of the second ovarian pedicle; LC = time of the ligature of uterine body just cranial to the cervix; SP = suture of the peritoneum; SC = skin suturing. Values (HR, RR, SAP, MAP, DAP, EtCO_2_) were expressed with median **(bold)** and range and (SpO_2_; T) with mean +/- standard deviation.

*Significant difference compared to baseline values

^†^ Significant difference between groups.

All animals spontaneously breathed. Significant bradypnea was recorded all time points in both groups (p = 0.005). Respiratory rate was significantly lower in the group B compared to the group A at 5 minutes after administration of tiletamine/zolazepam combined with acepromazine (p = 0.000).

Systolic arterial pressure (SAP) significantly decreased throughout the timeline in group A (p = 0.005). SAP significantly decreased, in group B, at the following time points (SD; IC; TPII; LC; SP; SC) (p = 0.000). In the group B, SAP was significantly higher, compared to the group A at B, SD and I times.

During surgery, (IC; TPI; TPII; LC; SD), SAP values were superimposable in the two groups except at the time of skin suturing (SC), in which the SAP values was significantly lower in the group B (p = 0.000).

MAP significantly increased in the group A. In the group B, MAP significantly increased after endotracheal intubation, at the time of the skin incision, during the traction of both ovarian pedicles, and at the time of the ligature of the uterine body, whereas MAP significantly decreased at 5 minutes after administration of tiletamine/zolazepam combined with acepromazine, at the beginning of the suture of the peritoneum and at the skin suturing. MAP was significantly higher in the group A compared to the group B at the time of the ligature of the uterine body, at the beginning of the suture of the peritoneum, and at the skin suturing (p = 0.000). MAP was significantly lower in the group A compared to the group B at 5 minutes after administration of tiletamine/zolazepam combined with acepromazine, after endotracheal intubation, and at the time of the skin incision.

DAP significantly decreased in the group B (except after endotracheal intubation), whereas DAP showed mild but significant changes in the group A. DAP was significantly lower in the group A compared to the group B at 5 minutes after administration of tiletamine/zolazepam combined with acepromazine, after endotracheal intubation, at the time of the skin incision and during the traction of the first ovarian pedicle. DAP was significantly higher in the group A compared to the group B during the traction of the second ovarian pedicle, at the time of the ligature of the uterine body, at the beginning of the suture of the peritoneum, and at the skin suturing (p = 0.000) [Table 1].

Significant hypercapnia was recorded at the ligature of the uterine body (LC), at the beginning of the suture of the peritoneum (SP), and at the skin suturing (SC) in both groups.

SpO_2_ remained within physiological range in both groups.

Body temperature remained within physiological range in both groups. Mild reduction in body temperature was recorded during the traction of the second ovarian pedicle, at the time of the ligature of the uterine body, at the beginning of the suture of the peritoneum, and at the skin suturing in both groups.

In group A concentration of inspired isoflurane significantly decreased compared to endotracheal intubation: during traction of the second ovarian pedicles (p = 0.000); at time of the ligature of the uterine body (p = 0.000); at beginning of the suture of the peritoneum (p = 0.000) and at skin suturing (p = 0.002). The concentration of inspired isoflurane, in the group B, decreased throughout the surgery, compared to the values after endotracheal intubation (p = 0.000) [Table 2].

**Table 2:** Concentration of inspired (CI) and expired (CE) isoflurane.

		Time						
Variables	Group	I	IC	TPI	TPII	LC	SP	SC
CI (%)	A B	2.3±0.1† 3±0	2.3±0.1*† 2.1±0.3*	2.4±0.1*† 1.7±1*	2±0.1*† 1.8±0.1*	2±0.2*† 1.7±0.1*	2±0.1*† 1.6±0.1*	1.5±1*† 1.6±1*
CE (%)	A B	1.5±0.2† 1.1±0.1	1.5±0.1 1.5±0.1*	1.3±0.1*† 1.4±0.1*	1.3±0.1*† 1.2±0.1*	1.4±0.1* 1.4±0.2*	1.4±0.1† 1.2±0.1*	1.3±0.1*† 1.1±0.1

Inspired (CI) and expired (CE) isoflurane. I = after endotracheal intubation; IC = skin incision; TPI = traction of the first ovarian pedicle (right); TPII = traction of the second ovarian pedicle; LC = time of the ligature of uterine body just cranial to the cervix; SP = suture of the peritoneum; SC = skin suturing. Values were expressed with mean and standard deviation (+/-)

*Significant difference compared to baseline values

†Significant difference between groups.

The concentration of inspired isoflurane was significantly lower in the group B compared to the group A throughout the surgery: at the skin incision (p = 0.04); during traction of the first ovarian pedicle (p = 0.03); during traction of the second ovarian pedicle (p = 0.001); at the times of the ligature of the uterine body (p = 0.001), at suture of the peritoneum (p = 0.001) and skin suturing (p = 0.001).

The concentration of inspired isoflurane was significantly lower in the group B compared to the group A after endotracheal intubation, during skin incision, the traction of ovarian pedicles at the beginning of the suture of the peritoneum, and at the skin suturing: (p = 0.000) [Table 2].

The CPS [Table 3] was significantly higher in the group A compared to the group B after endotracheal intubation, at the skin incision, and during the traction of the first ovarian pedicle (p = 0.000).

**Table 3:** Cumulative pain score (CPS).

	Time						
Groups	I	IC	TPI	TPII	LC	SP	SC
A B	2 (2–2) † 0 (0–0)	2 (2–2) † 0 (0–0)	1 (1–1) † 0 (0–0)	0 (0–0) 0 (0–0)	0 (0–0) 0 (0–0)	0 (0–0) 0 (0–0)	0 (0–0) 0 (0–0)

No dogs required rescue analgesia. Following tramadol administration, two dogs from group A displayed transient muscle twitching.

I = after endotracheal intubation; IC = skin incision; TPI = traction of the first ovarian pedicle (right); TPII = traction of the second ovarian pedicle; LC = time of the ligature of uterine body just cranial to the cervix; SP = suture of the peritoneum; SC = skin suturing. Values are median and range.

† Significant difference between groups (p<0.05).

## Discussion

This study shows that the administration of 1.5 mg/kg of tramadol followed by a CRI of 2.6 mg/kg/h of tramadol compared with the administration of a single IV bolus of 4 mg/kg of tramadol in dogs undergoing ovariohysterectomy reduces isoflurane requirements, and induces bradypnea and mild but significant reduction of heart rate and body temperature.

Tachycardia combined with a decrease in blood pressure was recorded after the administration of tiletamine/zolazepam and acepromazine, these changes could be explained by the pharmacological characteristics of these drugs [9,10]. Dissociative anesthetics combined with benzodiazepines provide a rapid induction of anesthesia and a good muscle relaxation. Nevertheless, this pharmacological mixture induces a significant bradycardia and consequent reduction in blood pressure both in dogs and cats [11]. Acepromazine is a neuroleptic drug which induces hypotension usually combined with consequent tachycardia. The hypotensive effect of acepromazine is related to both the dose and route of administration. Although hypotension produced by acepromazine is dose-dependent, a mild hypotension can occur even with very low intravenous doses [10].

Tramadol administration induced bradycardia and decrease in blood pressure. The effects of tramadol on HR and blood pressure are related to species, anesthetic management, and tramadol dose and route of administration. Tramadol induces tachycardia and hypertension in rabbits anesthetized with urethane and α-chloralose [12], and in rats anesthetized with intraperitoneal injection of pentobarbital [13]. On the contrary, tramadol induces a transient bradycardia in rabbits anesthetized with isoflurane [14]. Tramadol does not significantly reduce HR and blood pressure in dogs [2,6,7]. Nevertheless, tramadol produces a prolonged peripheral vasoconstriction in dogs anesthetized with sevoflurane coupled with a transient and mild hypertension [6]. We cannot affirm with certainty that changes in HR and blood pressure along the time line are related to tramadol administration. It is likely that these changes may also be due to the isoflurane effects on cardiac hemodynamics [6,7].

Bradypnea may be due to the side effects of tiletamine/zolazepam [3,4,11]. Tramadol does not cause respiratory depression and usually induces tachypnea in anesthetized dogs and horse [2]. However, we cannot exclude that the respiratory depression may be related to an additive depressive effect of tramadol. This disadvantage could be overcome by reducing the dose of CRI of tramadol [1]. Bradypnea may also be due to the isoflurane because inhalation anesthetics lead to dose-related cardiorespiratory depression. Hypercapnia was probably due to the observed respiratory depression. Despite bradycardia and bradypnea, SpO_2_ values showed a good tissue perfusion and oxygenation in both groups.

Hypothermia during the perioperative period is usually multifactorial with contributions from the anesthetic, surgical exposure, and patient factors. It is likely that the recorded mild reduction of body temperature may be related to evaporative heat loss through the surgical wound [15].

Dogs received tramadol as a CRI required lower isoflurane concentrations compared to dogs received a single IV bolus of tramadol. The reduction in isoflurane requirements is a key point for the anesthetist because it results in reduced cardiorespiratory depression and cost saving. Our results are consistent with those reported previously: both a single intravenous dose of tramadol and a CRI of tramadol have a significant effect on reducing isoflurane and sevoflurane requirement in experimental models [2,6,16]. Some factors influence isoflurane or sevoflurane requirement including species, age, surgical procedure, drug used, healthy status, changes in body temperature, severe hypotension, and acidemia [7,17]. We enrolled in the present study only young and healthy dogs to minimize individual variability as a source of error.

Although the time period for the surgical procedure was short in both groups, dogs which were given a CRI of tramadol experienced less pain [18]. It is likely that a CRI of tramadol assures a constant level of analgesia [2]. However, fentanyl as rescue analgesia has been administered in no dogs. This was expected because the analgesic efficacy of tramadol has been previously demonstrated in dogs undergoing electric noxious stimulus subcutaneously [2]. The excellent analgesic effect of tramadol is related to a μ-opioid receptor affinity coupled with inhibitions of synaptic reuptake of monoamine neurotransmitters [5]. Consequently, preemptive administration of tramadol may also be useful for postoperative pain management of dogs undergoing ovariohysterectomy [6].

Muscle twitching was recorded in two dogs that were given tramadol as an IV single bolus, whereas this side effect was not recorded in dogs which were administered tramadol as a CRI.

This finding is consistent with previous studies in rat, horse and cattle in which excitement, tremors and muscle twitching are usually dose-related [3,4,19–21].

This study has some limitations in fact it would be appropriate to investigate the use of tramadol in CRI on surgical procedures that require longer time.

Tramadol may be particularly useful in dogs with poor cardiopulmonary function, with impaired hepatic or renal function, and in dogs in which nonsteroidal anti-inflammatory drugs are not recommended. However, observations of this study were solely obtained using healthy individuals that subjected to ovariohysterectomy and we cannot assert that tramadol is safe even on patients considered less stable.

Further investigation will be necessary to confirm the sparing effect of tramadol on minimum alveolar concentrations of isoflurane in dogs undergoing ovariohysterectomy. It is known that the plasma concentration of tramadol gradually decreases over time but no data are available about the minimum effective plasma concentrations of tramadol in dogs [2,5–7].

## Supporting information

S1 Dataset (XLSX)
